# Prognostic factors and survival of patients with melanoma treated at a reference hospital in the Brazilian Amazon region^☆^

**DOI:** 10.1016/j.abd.2022.12.013

**Published:** 2024-05-01

**Authors:** Amanda Gabay Moreira, Antonio Vitor da Silva Freitas, Carla Andrea Avelar Pires

**Affiliations:** a Department of Dermatology, Universidade do Estado do Pará, Belém, PA, Brazil; b Department of Medicine, Universidade do Estado do Pará, Belém, PA, Brazil

Dear Editor,

Melanoma is responsible for 90% of skin cancer deaths. In Brazil, in 2020, 4,250 new cases were recorded in women and 4,200 in men, with the northern region accounting for 190 new cases, and the state of Pará, for 26.50% of these.^1^

There are risk factors to be considered for melanomas, such as numerous dysplastic nevi, family history, and individuals with lower phototypes on the Fitzpatrick scale.^2^ The five-year survival rate for patients with *in situ* melanoma is greater than 90%, while for those with regional disease and distant metastases it is 62% and 16%, respectively.^1^, ^2^

The objective of the study is to describe and correlate the epidemiological and clinical profile of patients with primary cutaneous melanoma at Hospital Ophir Loyola, a reference center for cancer in the Brazilian Amazon region. Additionally, the aim was to classify the types of melanomas and correlate them with the presence of metastasis, analyzing Clark level and Breslow thickness, identify patient survival time and correlate it with the aforementioned parameters and the presence of compromised lymph nodes.

Data were collected retrospectively. The G test was used to assess the presence of metastasis and the survival curves were generated for survival analysis according to the Kaplan-Meier method for the total sample and using the Log-Rank test to compare curves, with the Hazard ratio being calculated considering gender, age, phototype and Clark level. The study was approved by the Research Ethics Committee, under counsel number 5,156,829.

Ninety-one medical records were evaluated, related to the period of January 2015 to December 2020, which had their epidemiological profile classified according to gender, age group and phototype. When analyzing clinical data, significance (p < 0.05) was observed both in the evaluation of melanoma types and in the classification of Clark levels and Breslow thickness, when correlated with the presence or absence of metastases (Table 1). Thirty-one patients had metastases, the main sites being the central nervous system, pulmonary system, and soft tissue, respectively. The overall survival (Fig. 1) of patients diagnosed with melanoma was assessed, as well as patient survival according to the Breslow thickness (Fig. 2) and in those with or without the presence of compromised lymph nodes (Fig. 3).

**Table 1:** Clinical characteristics in the presence or absence of metastases in melanoma patients treated at a reference service, Belém – Pará, 2015 to 2020.

Clinical characteristics	With metastasis		Without metastasis		p-value
	n	%	n	%	
**Gender**					
Male	17	54.84	24	40.00	Teste G
Female	14	45.16	36	60.00	0.2606
Total	31	100.00	60	100.00	
**Age range**					
20 to 30 years	‒	‒	2	3.33	G Test
31 to 40 years	1	3.23	4	6.67	0.4307
41 to 50 years	7	22.58	8	13.33	
51 to 60 years	9	29.03	16	26.67	
61 to 70 years	5	16.13	8	13.33	
71 to 80 years	7	22.58	10	16.67	
More than 80 years	2	6.45	12	20.00	
Total	31	100.00	60	100.00	
**Phototype**					
I	5	16.13	2	3.33	G Test
II	4	12.90	7	11.67	0.3050
III	9	29.03	15	25.00	
IV	8	25.81	21	35.00	
V	‒	‒	1	1.67	
VI	‒	‒	2	3.33	
No information	5	16.13	12	20.00	
Total	31	100.00	60	100.00	
**Type of melanoma**					
Acral lentiginous	10	32.26	17	28.33	Teste G
Superficial spreading	2	6.45	17	28.33	0.0268
Lentigo maligna	2	6.45	11	18.33	
Nodular	13	41.94	15	25.00	
No information	4	12.90	‒	‒	
Total	31	100.00	60	100.00	
**Clark level**					
1	‒	0.00	9	15.00	Teste G
2	‒	0.00	8	13.33	0.0001
3	3	9.68	12	20.00	
4	12	38.71	21	35.00	
5	16	51.61	10	16.67	
Total	31	100.00	60	100.00	
**Breslow thickness**					
Less than 1 mm	‒	0.00	17	28.33	Teste G
1 to 2 mm	3	9.68	13	21.67	<0.0001
3 to 4 mm	2	6.45	8	13.33	
More than 4 mm	26	83.87	22	36.67	
Total	31	100.00	60	100.00

**Figure 1:**
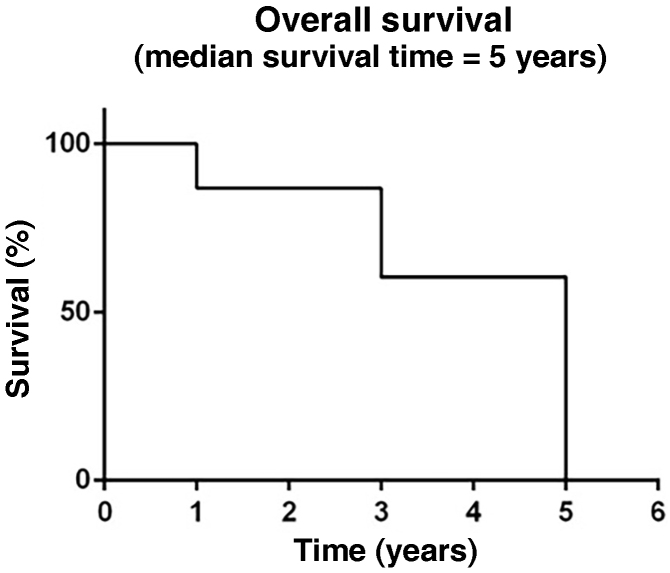
Overall survival of melanoma patients treated at a reference service, Belém – Pará, 2015 to 2020.

**Figure 2:**
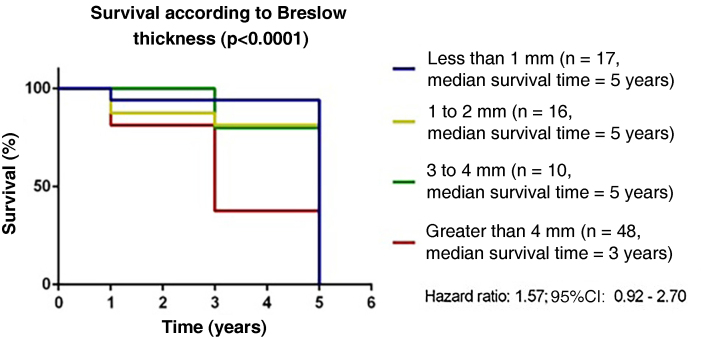
Survival according to Breslow thickness in melanoma patients treated at a reference service, Belém – Pará, 2015 to 2020.

**Figure 3:**
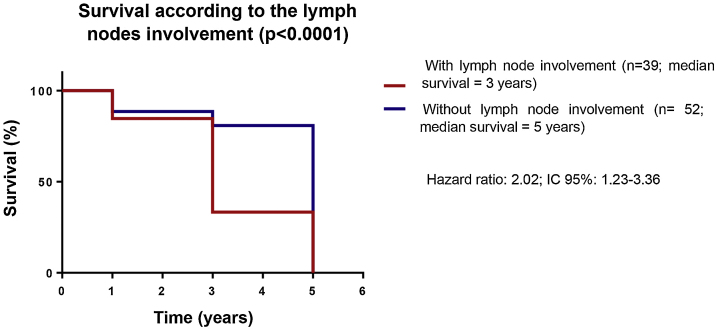
Survival according to the presence of lymph node involvement in melanoma patients treated at a reference service, Belém – Pará, 2015 to 2020.

After the data were evaluated, a slight predominance was identified in those with phototypes III and IV (p < 0.0001). These data differ from those in the literature, as the latter points to a higher incidence of melanoma skin cancer in patients with lighter phototypes, such as phototypes I and II, due to their lower ability to tan and a greater propensity to burn. It is worth highlighting that the phototypes described as the most frequent are those with the highest prevalence in the North region of the country, as described in the 2010 Brazilian census, which makes it a bias for the exclusive analysis of this aspect as a risk factor since it does not exclude the disease from occurring.^3^

Regarding the age group, it can be observed there is an agreement between increase in diagnoses and advancing age, with 50% of the assessed patients over 60 years old, corroborating the literature. The latter indicates age as an important risk factor for the occurrence and mortality of melanoma, due to excessive sun exposure and cumulative skin damage, sometimes associated with dysplastic nevi.^1^, ^3^

There was a higher prevalence of the nodular subtype, as well as its association with the presence of metastasis, in 42% of cases. These data confirm those in the literature, as these tumors are characterized by their rapid growth and high mortality rate, being responsible for approximately 40% of deaths from melanomas, classifying one of the most aggressive subtypes.^4^ This type of lesion does not have a recognizable radial growth phase, which makes it difficult to detect in the early stages.^5^ The acral lentiginous subtype was the second subtype with the highest association with metastasis (32.26%). This fact is justified by its histopathological characteristics, as this subtype, when compared to the superficial spreading,^6^ shows predictive factors for worse prognosis, such as a greater number of macrophages in peritumoral and intratumoral areas, which influence tumor thickness, ulceration, mitosis rate and metastasis.^7^

Statistical significance was observed for the presence of metastasis in more than half of the patients in which the tumor reached > 4 mm in thickness or Clark level IV (p = 0.0001), as well as with involvement of the subcutaneous tissue or Clark level 5 (p = 0.0001). This correlation is supported by current literature, as it shows that the greater the thickness and extension into adjacent tissues, the greater the chance of metastasis.^8^

Patient survival is influenced by several factors that lead to a worse prognosis, from general characteristics such as age to tumor subtype and mitotic activity.^9^ It was verified that less than 50% of patients with tumor thickness > 4 mm (p = 0.0001) involving the subcutaneous tissue (p = 0.0004) achieved a five-year survival. Breslow thickness is considered the most important factor for the occurrence of metastases, directly associated with the mitotic index of the neoplasm.^10^

Moreover, patients with involved lymph nodes had a mean survival of only three years after the diagnosis. This is a consistent result, as a lower survival rate can be expected in patients with an indication for sentinel lymph node biopsy, as they are risk factors for shorter disease-free time, such as Breslow thickness ≥ 1 mm and mitotic index ≥ 5/mm^2^.^8^ It is important to highlight that 74 patients in this study met these criteria and were then submitted to sentinel lymph node screening. Finally, even with the high prevalence of more invasive subtypes and the presence of worse prognostic factors, more than 50% of the patients achieved a five-year survival in the reference service.

This study, therefore, corroborates the clinical and epidemiological differences in the various services in the country, as well as promotes new hypotheses and research focused on the area of oncological dermatology. It is especially important to highlight, due to the high frequency of nodular and acral forms with Breslow thickness > 4 mm, the importance of raising awareness among health professionals and developing primary prevention campaigns, given that these types are responsible for 33% of metastases during follow-up. Therefore a more agile and effective health system flow is needed.

The main limitations of the study were the small sample size and, undoubtedly, access to data, as these were obtained from physical records, most of which lacking information on staging, with the analysis carried out by the researchers with the help of attached exams.

## Financial support

None declared.

## Authors’ contributions

Amanda Gabay Moreira: Critical review of the literature; collection, analysis and interpretation of data; statistical analysis; approval of the final version of the manuscript; design and planning of the study; drafting and editing of the manuscript; critical review of the manuscript.

Antônio Vitor Da Silva Freitas: Critical review of the literature; collection, analysis and interpretation of data; statistical analysis; approval of the final version of the manuscript; design and planning of the study; drafting and editing of the manuscript; critical review of the manuscript.

Carla Andrea Avelar Pires: Approval of the final version of the manuscript; design and planning of the study; effective participation in research orientation; critical review of the manuscript.

## Conflicts of interest

None declared.
